# Exposure to Traumatic Events and Shame in Adolescent Surf Lifesavers: An Australian Perspective

**DOI:** 10.1007/s40653-024-00662-1

**Published:** 2024-10-10

**Authors:** Samantha Fien, Jasmin C. Lawes, Jessica Ledger, Ian de Terte, Murray Drummond, Pamela Simon, Nancy Joseph, Shane Daw, Sean Kelly, Wendy Hillman, Robert Stanton, Talitha Best

**Affiliations:** 1 School of Health, Medical and Applied Sciences, Central Queensland University, Mackay, QLD Australia; 2 Research Cluster for Resilience and Wellbeing, Appleton Institute, Wayville, South Australia Australia; 3 Surf Life Saving Australia, Bondi Beach, Sydney, NSW Australia; 4 School of Psychology, Massey University, Wellington, New Zealand; 5 Flinders Institute for Mental Health and Wellbeing (FIMHWell), Flinders University, Adelaide, South Australia Australia; 6 Sport, Health, Activity, Performance and Exercise Research Centre, Flinders University, Adelaide, South Australia Australia; 7 School of Health, Medical and Applied Sciences, Central Queensland University, Rockhampton, Queensland Australia; 8 School of Health, Medical and Applied Sciences, NeuroHealth Lab, Appleton Institute, Central Queensland University, Brisbane, Queensland Australia

**Keywords:** Emergency response, Lifesaving, Mental health, Shame, Trauma, Volunteers

## Abstract

Emergency service personnel experience high levels of psychological distress, with increasing evidence of associations with shame and trauma. Additionally, adolescence is a critical time in social and cognitive development, in which shame plays an important role. In Australia, adolescent volunteer surf lifesavers (SLS) are particularly vulnerable due to exposure to potentially traumatic experiences (PTEs) such as rescues and resuscitation of human lives. The aim of this study was to investigate the association between direct or indirect PTEs, and the relationship PTSS and shame may have in adolescent surf lifesavers. This cross-sectional study surveyed patrolling adolescent SLS, aged 13–17 years, recruited via internal communications and social media groups. Complete responses from patrolling adolescents (*n* = 118; 59% female; mean age 15.4 years) were used to determine exposure to PTEs across global, direct, and within SLS trauma domains. Associations between demographics, PTEs, post-traumatic stress symptoms (PTSS), with shame as a moderator were assessed for each trauma domain. PTEs and PTSS were positively associated across trauma domains. Shame was identified as a significant predictor of PTSS and as an important moderator of PTSS for experiences within SLSA, but not global or direct trauma. By exploring links between PTEs, PTSS, and shame, these findings contribute to the development of strategies and interventions for adolescents during stressful times. Responsiveness to adolescents via feedback and genuine, reassuring relationships that acknowledge the complexity of coping with stressful situations, may be potentially effective approaches to support coping with experiences of shame following PTE’s in adolescent surf lifesavers.

## Introduction

Emergency service personnel experience higher psychological distress and mental ill health compared to the general population (Beyond Blue Ltd., 2018; Education and Employment References Committee, 2019). Surf Life Saving Australia (SLSA) is a unique emergency response organisation worth $6.5 billion to the Australian community annually (Deloitte., 2020). SLSA membership was approximately 190,000 in 2022, with 44,272 (23%) who volunteer their time providing beach patrols, and emergency and support services (Surf Life Saving Australia., 2021).

By providing these services, members may be at risk of potentially traumatic events (PTEs) as they assist, treat, and provide vital aid to the public (Fien et al., 2021). Importantly, SLSA volunteers can patrol from as young as 13 years of age and adolescents between 13 and 17 years of age may be directly or indirectly exposed to a range of PTEs. To date, the experience of adolescent SLSA volunteers and their experience of PTE’s and stress responses has not been explored (Fien et al., 2021).

As a developmental period, adolescence is a time of the highest risk of exposure to PTEs (Finkelhor et al., 2009; Wetterlöv et al., 2021) and prevalence of PTEs among adolescents ranges from 61.8 to 84.1% (Aho et al., 2016; McLaughlin et al., 2013). Exposure to both direct and indirect PTEs may lead to subsequent mental health difficulties in adolescents (Bödvarsdóttir & Elklit, 2007). For young surf lifesavers (aged 17–26 years), preliminary findings indicate they are more vulnerable to symptoms of PTSS compared to older lifeguards (Rooke & de Terte, 2024). To date, however, there are no studies of adolescent surf lifesavers’ exposure to PTEs and subsequent psychological difficulties, such as PTSS.

Adolescence is a critical time in social and cognitive development and in defining social identity, roles, and peer-relationships (Ragelienė, 2016). Shame, as a self-conscious emotion, plays an important role in social functioning and coping. Broadly, while there are diverse interpretations on the definition of shame, and in particular post-traumatic shame, there is general consensus by clinicians, theorists, and researchers that shame is both internally and externally based (DeCou et al., 2023) and involves the appraisal of an individual’s characteristics and behaviour in their own mind (self) and in the mind of another (other). Shame is usually conceived as a painful emotional experience and attribution of the self as unattractive, undesirable, worthless, inferior, or defective in some way, and associated with having a failure or flaw exposed (Gilbert & Irons, 2008; Irons & Gilbert, 2005). External shame refers to the persistent and overwhelming perception that others hold negative beliefs and thoughts about one’s self (Cunha et al., 2012). In contrast, internal shame refers to holding a negative and persistent perception about oneself that leads to submissive behaviour, withdrawal, self-criticism, and feelings of inferiority (Garnefski et al., 2005; Gilbert & Irons, 2008; Pinto-Gouveia & Matos, 2011).

Shame is a common experience and can be adaptive in developing awareness of prosocial behaviours and lower levels of aggression to guide one’s interactions with others (Gilbert, 2017; Mills, 2005). However, shame can also be detrimental and in adolescents has been linked with anxiety and depression (Cunha et al., 2012), eating disorders, delinquent behaviours, and substance use (Rahim & Patton, 2015). During adolescence, individuals become more aware of peer relationships, develop reasoning processes, form connections between the self and past events, and become more aware of how they exist in the minds of others which makes them more vulnerable to the emotional impact of shame (Cunha et al., 2012). Consequently, shame is a significant risk factor for the onset and continuation of mental health difficulties in adolescents (Paulo, Vagos, Ribeiro Da Silva, & Rijo, 2020). Moreover, shame related to PTEs in childhood and adulthood has been related to psychological difficulties (DeCou et al., 2023; Dyer et al., 2017).

Understanding the association between direct or indirect PTEs, in adolescent surf lifesavers and the relationship to PTSS is important for understanding risk factors of shame to reduce the mental health vulnerability of adolescents in this volunteer role. The research that underpins this paper is essential to SLSA and other emergency services, clinicians, and counsellors to understand adolescent experience of PTEs, PTSS, and shame. We hypothesise that adolescent surf lifesavers with greater exposure to PTEs will report higher PTSS, with shame (external and internal) moderating the relationship between PTEs and PTSS. From a clinical perspective, outcome of this research may be applied to the treatment and prevention of psychological problems in adolescents.

## Method

An online, anonymous adolescent survey was used based on previously piloted research conducted within the timeframe of October 2020 - January 2021 (Fien et al., 2023). The study was approved by Central Queensland University Human Research Ethics Committee (HREC 22265). Data were collected using the Qualtrics online survey platform, whereby informed consent was required by the participant and the parental/legal guardian of the participant.

### Participants

Participants were recruited through the SLS membership database via email and specific SLS private Facebook group pages. The survey was distributed to adolescent members aged 13–17 years during May to August 2021.

### Completion Rates

In total, 276 adolescents responded to the survey with 118 complete responses included in analysis, indicating a completion rate of 43%. Incomplete responses included, *N* = 22 after initial question of consent, *N* = 82 after the demographics, and *N* = 13 after completing 53–79% of survey. The 95 participants that commenced survey items were predominately female (62.11%), with an average age of 15 years (M = 15.64, SD = 1.34), were born in Australia (95.79%,) and did not identify as Aboriginal and/or Torres Strait Islander.

The high attrition after completing the demographic items may be due to the length of the survey and it’s attentional and time demand in an adolescents’ daily life.

### Measures

#### Demographics

Participants were asked eight items related to gender, age, membership role in SLS, the state in which their club was located, years as a member of SLS, and years spent as an active patrolling member.

#### Adolescent Life Events

Life events were measured using the following: five items from Life Events Checklist (LEC-5; Cronbach’s alpha = 0.79 which were piloted to include for the adolescent population as they related to the role of lifesaver (e.g., natural disasters) (Fien et al., 2023), 39 items from the Adolescent Life Events Stress Scale (ALESS; (Aggarwal, 2007), seven SLS specific items (Rooke & de Terte, 2024), one item related to the COVID-19 pandemic (due to the situation and effect in Australia at the time of the survey being released), followed by a final item used to screen for any other PTE not otherwise specified. An additional six SLS-specific questions were developed and included: drowning fatality, providing treatment and/or support to a significant incident, responsible for filling out forms (e.g., incident forms), handover to emergency services (e.g., police, paramedics), dealing with self-harm incidents, and dealing with someone under intoxication (e.g., alcohol, illegal drugs, prescribed medications). In total, 59 items were included, with response options for each domain shown in Table 1.

**Table 1:** Trauma domains

Trauma	Definition	ALESS responses included in score
Global Trauma	Trauma the participant has either experienced, witnessed, or learnt about	1 = Happened to me, 2 = Witnessed it, 3 = Learned about it
Direct Trauma	Trauma experienced by the participant	1 = Happened to me
Trauma within SLS	Trauma that has occurred within SLS role	1 = Happened to me, OR 2 = Witnessed it, AND 4 = Within SLS

ALESS = Adolescent Life Events Stress Scale; SLS = surf lifesaving

#### Post-Traumatic Stress

The 20-item Post-Traumatic Stress Disorder Checklist-5 (PCL-5) measures severity of PTSS experienced within the past month (Weathers et al., 2013), and has been validated in adolescents as young as 7 years old (Lin et al., 2021). Each symptom is rated on a five-point scale ranging from “0 = Not at all, 1 = A little bit, 2 = Moderately, 3 = Quite a bit, 4 = Extremely”. Items are summed to provide a total severity score (0–80): higher scores indicate more severe PTSS. A score of ≥ 33 indicates PTSS, but a confirmed diagnosis of PTSD can only be made by a clinician (Weathers et al., 2013).

#### Shame

Two subscales from The Attitude Towards Mental Health Problems scale (ATMHP; Cronbach’s alpha = 0.85; (Gilbert et al., 2007) were used to explore external and internal shame. Items were measured on a four-point scale ranging from “1 = Do not agree at all, 2 = Agree a little, 3 = Mostly agree, 4 = Completely agree” to provide a total score for each subscale. External shame had a total of 10 items focussing on the perception of how their SLS community (5 items) and family (5 items) would perceive them if they had a mental health problem, respectively. The internal shame subscale had 5 items focussing on how an individual perceives themself if they had a mental health problem. Higher scores on each scale indicated greater external shame and internal shame (Cronbach’s alpha inter-item correlation for subscales: external shame = 0.85 and internal shame = 0.57).

### Statistical Analyses

All analyses were performed using SPSS 27 (IBM Corp, Armonk, NY). All *p* values were 2-sided with demographic characteristics reported as *M (SD)* or *n*/%. Bivariate correlations were used to assess the relationships between age, stressful life events, and PTSS. Hierarchical regression analyses were used to explore effects of significantly correlated predictor variables on PTSS for each trauma domain measured (i.e., global trauma, direct trauma, and trauma within SLS; Table 1). A hierarchical multiple regression was completed for each trauma domain to determine the contribution of variables to PTSS, and the effects of external and internal shame measures. Demographic variables were accounted for in the first step of the model, with internal and then external shame in following models to determine the variation explained by each shame measure. To reduce impacts of small sample sizes of non-binary gender and prefer not to disclose gender categories, only responses from males and females were included in these analyses. Multicollinearity and tolerance assumptions for hierarchical regression analyses were checked and met. The moderating roles of internal and external shame on the relationship between PTSS on each trauma domain were then tested using two-step regression analyses and the creation of an interaction variable as described by Hayes (2013).

## Results

### Survey Sample Characteristics

Over half the sample identified as female (58.5%, *n* = 69; Table 2), and the mean age of participants was 15.4 years (*SD* = 1.3; Table 3). Participants had been members of SLS for an average of 5.9 years (*SD* = 3.5) and had been patrolling for an average of 2.5 years (*SD* = 1.3; Table 3). Most participants were born in Australia (89.8%, *n* = 106; Table 2) with three-quarters from Queensland (75.4%, *n* = 89; Table 2). The criteria for PTSS was met by 39 respondents (33%; Table 2) of sample.

**Table 2:** Survey demographics

Variable	*n*	%
Respondents (100% completion)	118	100
Gender		
Male	42	35.6
Female	69	58.5
Genderqueer/Non-binary	4	3.4
Prefer not to disclose	3	2.5
Birth Country		
Australia	106	89.8
Other	12	10.2
Indigenous		
Yes	9	8.6
No	109	92.4
State		
Queensland	89	75.4
New South Wales	18	15.3
Victoria	3	2.5
Western Australia	5	4.2
South Australia	3	2.5
Role in Surf Life Saving ^*^		
A volunteer surf lifesaver	117	99.2
A lifeguard	7	5.9
Associate member^α^	7	5.9
Member Years^β^		
0–9	98	83.1
10–19	20	16.9
PTSS (PCL-5 scores)		
Indicates that the criteria for PTSS are met (PCL-5 score > 33)	39	33.1
Indicates that the criteria for PTSS are not met (PCL-5 score < 33)	79	66.9

PTSS = Post-traumatic stress symptoms

* = participants could choose more than one response for this question

α = associate member is someone who does not actively patrol but is associated with the organisation

β = participants could be a member within the organisation from as young as 5 years of age due to the Junior Activities Program which teaches essential surf skills, knowledge and education of the beach and cardiopulmonary resuscitation

### Correlations

The correlation matrix showed positive correlations between variables (Table 3). Many correlations were quite weak, with most of the strongest correlations observed between related variables (i.e., trauma domains and shame measures). In line with our hypotheses, PTSS correlated quite strongly and significantly with all three trauma domains: global trauma (*r* (116) = 0.33, *p* < .001), direct trauma (*r* (116) = 0.54, *p* < .001), and trauma within SLS events (*r* (116) = 0.35, *p* < .001). Global trauma was strongly correlated with direct trauma (*r* (116) = 0.50, *p* < .001) and trauma within SLS (*r* (116) = 0.57, *p* < .001), as direct trauma was correlated with trauma within SLS (*r* (116) = 0.50, *p* < .001). The strongest correlation was between PTSS and external shame (*r* (116) = 0.65, *p* < .001), with a similar relationship strength observed with internal shame (*r* (116) = 0.49, *p* < .001). Correlations between age and shame were very weak and not significant. External shame was significantly correlated with all three trauma domains, but with varying strengths: global trauma (*r* (116) = 0.22, *p* = .018), direct trauma (*r* (116) = 0.41, *p* < .001) and trauma within SLS events (*r* (116) = 0.40, *p* < .001), while internal shame was correlated with global trauma (*r* (116) = 0.23, *p* = .014) and direct trauma (*r* (116) = 0.31, *p* < .001) only. Relationships of PTSS and external shame measures with direct trauma and trauma within SLS were stronger than those with global trauma (Table 3).

**Table 3:** Descriptive statistics and Pearson’s correlations (Two-tailed) for continuous study variables

Variable	*n*	M	SD	1	2	3	4	5	6
1. Age	118	15.36	1.26	-					
2. Global Trauma	118	0.70	0.39	0.09	-				
3. Direct Trauma	118	0.29	0.14	0.14	0.50***	-			
4. Trauma within SLS	118	0.09	0.17	0.03	0.57***	0.50***	-		
5. PTSS (PCL-5)	118	23.97	19.76	0.25**	0.33***	0.54***	0.35***	-	
6. External Shame	118	16.46	7.92	0.11	0.22*	0.41***	0.40***	0.65***	-
7. Internal Shame	118	10.50	4.40	0.05	0.23*	0.31**	0.17	0.49***	0.55***

* *p* < .05, ** *p* < .01, *** *p* < .001

PTSS = Post-traumatic stress symptoms; SLS = surf lifesaving

### Hierarchical Regression Analyses of Predictors

Regression coefficients and standard errors can be found in Table 4.

#### Global Trauma

In Model 1 of the hierarchical multiple regression, age, gender, and global trauma each contributed significantly to the regression model, (*F*(3,107) = 7.10, *p* < .001), and accounted for 16.6% of the variation in PTSS scores. Model 2 explained an additional 19.7% of variation in PTSS scores and this change in R² was significant, (*ΔF*(1,106) = 32.86, *p* < .001), with age, global trauma, and internal shame each contributing significantly to the model. Finally, Model 3 explained an additional 14.8% of the variation in PTSS scores and this change in R² was also significant, (*ΔF*(1,105) = 31.82, *p* < .001). The full model of age, gender, global trauma, internal shame, and external shame (Model 3) was statistically significant (Table 4). When all five predictor variables were included into the final regression model, neither age nor gender were significant predictors of PTSS. The most significant predictor of PTSS was external shame which uniquely explained 23.2% of the variation in PTSS scores. Together the five independent variables accounted for 51.1% of the variance in PTSS scores (Table 4).

#### Direct Trauma

In Model 1 of the hierarchical multiple regression, age, gender, and direct trauma each contributed significantly to the regression model, (*F*(3,107) = 13.42 *p* < .001), and accounted for 27.3% of the variation in PTSS scores. Introducing the internal shame variable into Model 2 explained an additional 13.9% of variation in PTSS scores and this change in R² was significant, (*ΔF*(1,106) = 25.18, *p* < .001), with age, direct trauma, and internal shame each contributing significantly to the model. Finally, Model 3 explained an additional 12.1% of the variation in PTSS scores and this change in R² was also significant, (*ΔF*(1,105) = 27.28, *p* < .001). The full model of age, gender, direct trauma, internal shame, and external shame (Model 3) was statistically significant (Table 4). When all five predictor variables were included into the final regression model, age and gender were no longer significant predictors of PTSS. The most significant predictor of PTSS was external shame which uniquely explained 20.6% of the variation in PTSS scores. Together the five independent variables accounted for 53.4% of the variance in PTSS scores (Table 4).

#### Trauma within SLS

In Model 1 of the hierarchical multiple regression, age, gender, and trauma within SLS each contributed significantly to the regression model, (*F*(3,107) = 7.10, *p* < .001), and accounted for 16.6% of the variation in PTSS scores. Model 2 explained an additional 20.0% of variation in PTSS scores and this change in R² was significant, *(ΔF*(1,106) = 33.55, *p* < .001), with age, trauma within SLS, and internal shame each contributing significantly to the model. Finally, Model 3 explained an additional 12.1% of the variation in PTSS scores and this change in R² was also significant, (*ΔF*(1,105) = 24.69, *p* < .001). The full model of age, gender, trauma within SLS, internal shame, and external shame (Model 3) was statistically significant (Table 4). When all five predictor variables were included into the final regression model, gender, and trauma within SLS were no longer significant predictors of PTSS. The most significant predictor of PTSS was external shame which uniquely explained 19.0% of the variation in PTSS scores. Together the five independent variables accounted for 48.7% of the variance in PTSS scores (Table 4).

**Table 4:** Summary of hierarchical multiple regressions with PTSS as the outcome variable

Global Trauma									Direct Trauma									Trauma Within SLS								
Variable	β	B	SE	sr^2^	*R*	*R* ^2^	ΔR^2^	*p*	Variable	β	B	SE	sr^2^	*R*	*R* ^2^	ΔR^2^	*p*	Variable	β	B	SE	sr^2^	*R*	*R* ^2^	ΔR^2^	*p*
Model 1: F(3,107) = 7.100***					0.41	0.17	0.17	< 0.001***	Model 1: F(3,107) = 13.419***					0.52	0.27	0.27	< 0.001***	Model 1: F(3,107) = 7.103***					0.41	0.17	0.17	< 0.001***
Intercept		-35.46	20.98					0.094	Intercept		-37.84	19.59					0.056	Intercept		-32.42	20.94					0.125
Age	0.17	2.50	1.35	0.03				0.067	Age	0.15	2.32	1.26	0.03				0.068	Age	0.18	2.80	1.35	0.04				0.04*
Gender	0.14	5.41	3.48	0.02				0.123	Gender	0.10	3.81	3.27	0.01				0.488	Gender	0.15	5.67	3.48	0.02				0.106
Global Trauma	0.33	16.04	4.33	0.11				< 0.001***	Direct Trauma	0.47	65.89	11.72	0.23				< 0.001***	Trauma within SLS	0.33	37.25	10.1	0.11				< 0.001***
Model 2: F(4,106) = 15.126***					0.60	0.36	0.20	< 0.001***	Model 2: F(4,106) = 18.632***					0.64	0.41	0.14	< 0.001***	Model 2: F(4,106) = 15.336***					0.61	0.37	0.20	< 0.001***
Intercept		-46.46	18.52					0.014*	Intercept		-46.72	17.78					0.010*	Intercept		-44.63	18.45					0.017*
Age	0.16	2.49	1.19	0.04				0.038*	Age	0.16	2.37	1.14	0.04				0.040*	Age	0.18	2.68	1.18	0.05				0.025*
Gender	0.04	1.59	3.13	0.01				0.612	Gender	0.03	1.00	3.00	0.01				0.740	Gender	0.05	1.75	3.12	0.01				0.576
Global Trauma	0.21	10.22	3.93	0.06				0.011*	Direct Trauma	0.32	45.68	11.33	0.13				< 0.001***	Trauma within SLS	0.22	24.54	9.07	0.06				0.008**
Internal Shame	0.47	2.04	0.36	24				< 0.001***	Internal Shame	0.41	1.77	0.35	0.19				< 0.001***	Internal Shame	0.47	2.05	0.35	0.24				< 0.001***
Model 3: F(5,105) = 21.983***					0.73	0.49	0.15	< 0.001***	Model 3: F(5,105) = 24.058***					0.73	0.53	0.12	< 0.001***	Model 3: F(5,105) = 19.950***					0.70	0.49	0.12	< 0.001***
Intercept		16.31	16.31					0.008**	Intercept		-44.17	15.92					0.007**	Intercept		-42.28	16.7					0.013*
Age	0.13	1.05	1.05	0.03				0.062	Age	0.13	1.93	1.02	0.03				0.061	Age	0.14	2.16	1.08	0.04				0.047*
Gender	0.04	2.75	2.75	0.01				0.600	Gender	0.03	0.98	2.68	0.01				0.716	Gender	0.03	1.27	2.82	0.01				0.654
Global Trauma	0.18	3.47	3.47	0.05				0.016*	Direct Trauma	0.25	34.95	10.35	0.10				0.001**	Trauma within SLS	0.07	7.89	8.86	0.01				0.375
Internal Shame	0.20	0.38	0.38	0.05				0.027*	Internal Shame	0.18	0.76	0.37	0.04				0.043*	Internal Shame	0.23	1.01	0.38	0.06				0.010*
External Shame	0.48	0.21	0.21	0.23				< 0.001***	External Shame	0.44	1.09	0.21	0.21				< 0.001***	External Shame	0.47	1.15	0.23	0.19				< 0.001***

*Note: N* = 111 (Non-binary genders and those who did not disclose were excluded from these analyses, *n* = 7). *Β* = standardised regression coefficients. *B* and *SE* = unstandardised regression coefficients. *R* = multiple correlation coefficients. *R*^*2*^ = coefficients of determination. *ΔR*^*2*^ = *R*^*2*^ change. *sr*^*2*^ = squared semipartial correlation coefficients. * *p* < .05, ** *p* < .01, *** *p* < .001

PTSS = Post-traumatic stress symptoms; SLS = surf lifesaving

### Shame as a Moderator

#### Moderation Analysis of Shame with Global Trauma

In the first model, two variables were included: global trauma and shame. Shame accounted for a significant amount of variance in PTSS: both external shame, (*R*^*2*^ = 0.47, *F*(2,115) = 49.99, *p* < .001) and internal shame, (*R*^*2*^ = 0.30, *F*(2,115) = 24.09, *p* < .001).

When global trauma and shame were added to the regression models, the interaction term did not account for a significant portion of the variance of PTSS in any of the analyses: external shame, *(ΔR*^*2*^ = 0.01, *ΔF*(1, 114) = 2.94, *p* = .089, b = -0.73, *t*(114) = -1.71, *p* = .089), and internal shame, (*ΔR*^*2*^ = 0.01, *ΔF*(1, 114) = 1.13, *p* = .290, b = -1.02, *t*(114) = -1.06, *p* = .290). This finding indicates that the relationship between global trauma and PTSS was not moderated by shame.

#### Moderation Analysis of Shame with Direct Trauma

In the first model, two variables were included: direct trauma and shame. Shame accounted for a significant amount of variance in PTSS: external shame, (*R*^*2*^ = 0.52, *F*(2,115) = 61.92, *p* < .001), and internal shame, (*R*^*2*^ = 0.41, *F*(2,115) = 40.55, *p* < .001). When the interaction term between direct trauma and shame was added to the regression models, the interaction term did not account for a significant portion of the variance of PTSS in any of the other analyses; external shame, (*ΔR*^*2*^ = 0.01, *ΔF*(1, 114) = 3.37, *p* = .069, b = -1.74, *t*(114) = -1.84, *p* = .069), and internal shame, (*ΔR*^*2*^ = 0.00, *ΔF*(1, 114) = 0.46, *p* = .499, b = -1.53, *t*(114) = -0.68, *p* = .499). This finding indicates that the relationship between direct trauma and PTSS was not moderated by shame.

#### Moderation Analysis of Shame with Trauma within SLS

In the first model, two variables were included: trauma within SLS and shame. Shame accounted for a significant amount of variance in PTSS: external shame, (*R*^*2*^ = 0.44, *F*(2,115) = 44.42, *p* < .001), and internal shame, (*R*^*2*^ = 0.32, *F*(2,115) = 26.66, *p* < .001).

**Fig. 1:**
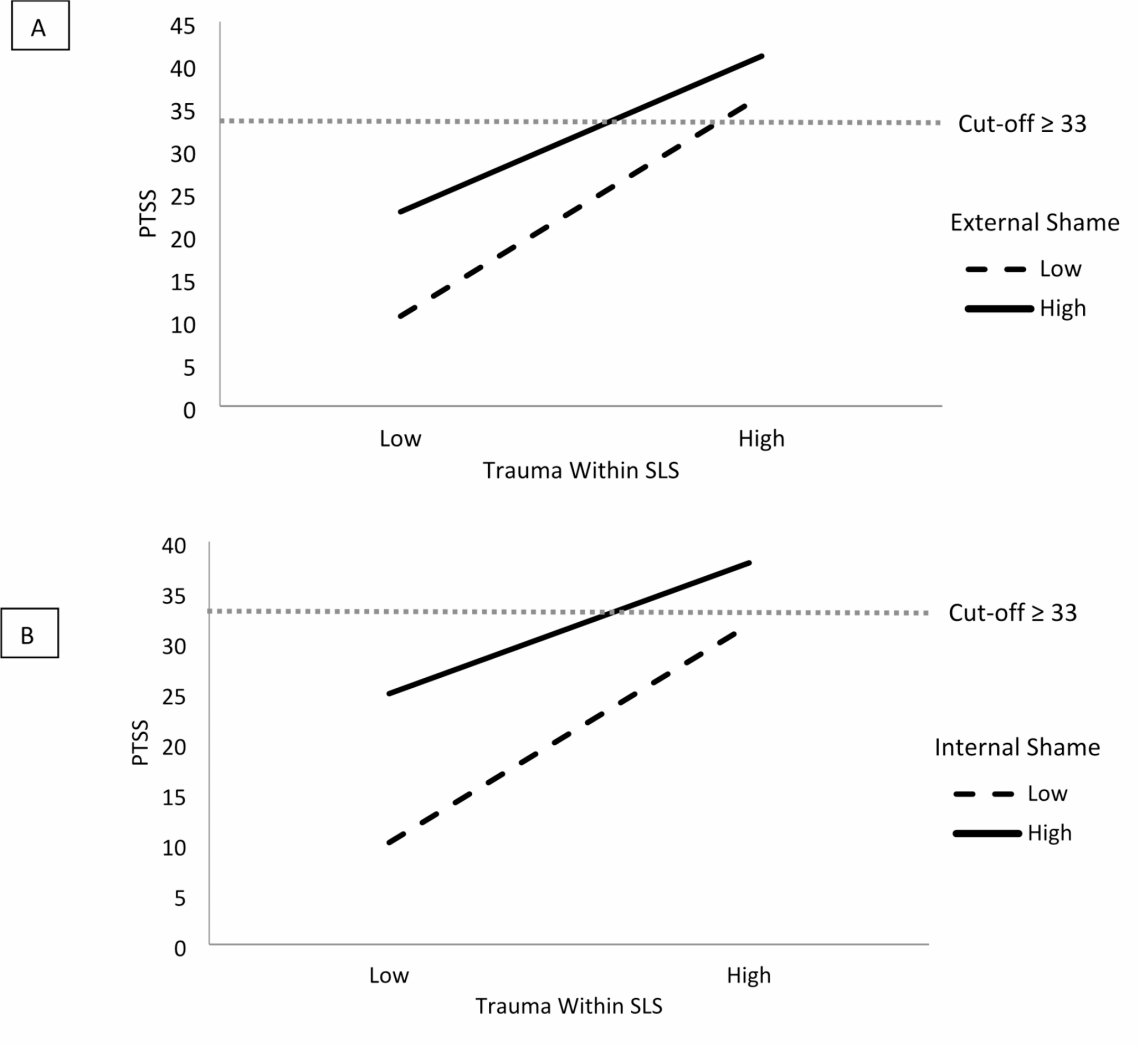
Interaction plots showing moderating effects of low and high (**A**) external and (**B**) internal shame on the relationship between PTSS and trauma within SLS. Low shame category denotes 1 SD below the mean, while High is 1 SD above the mean. Grey dotted line represents cut-off PTSS score indicating that the criteria for PTSS are met (PCL-5 score ≥ 33). PCL-5 = PTSD checklist; PTSS = post-traumatic stress symptoms; SD = standard deviation; SLS = Surf Life Saving

When the interaction terms of trauma within SLS and shame were added to the regression models, the interaction term accounted for a significant amount of the variance in PTSS severity for both external shame, (*ΔR*^*2*^ = 0.03, *ΔF*(1, 114) = 7.36, *p* = .008, b = -2.00, *t*(114) = -2.71, *p* = .008), and internal shame, (*ΔR*^*2*^ = 0.03, *ΔF*(1, 114) = 6.02, *p* = .016, b = -3.72, *t*(114) = -2.45, *p* = .016). This finding indicates that shame moderated the relationship between trauma within SLS and PTSS, such that when participants had lower (internal/external) shame then the relationship between trauma and PTSS was not as strong (Fig. 1).

## Discussion

This study addressed the PTEs of adolescent surf lifesavers and associations with PTSS and shame. In line with our hypotheses, exposure to PTEs was positively associated with greater PTSS. There was a strong predictive role of external and internal shame in PTSS across domains of PTE’s (i.e., global trauma, direct trauma, and trauma within SLS). Further, higher levels of shame (external and internal) were associated with higher levels of PTSS and were significant moderators of PTSS for trauma within SLS, but not for direct or global trauma.

Most individuals exposed to PTEs do not develop long-term outcomes such as PTSD or depression. Rather, the feelings and reaction to the traumatic experience, such as guilt, shame, or anger, may play a role in adjustment and recovery (Fletcher, 2011). Our findings suggest that SLSA adolescents who experience stressful situations can feel a sense of disconnection from those around them (external shame) and internalise feelings of inadequacy and insignificance (internal shame). Critically, our findings show that exposure to, or the experience of, a global traumatic event, a direct event, or an event experienced within SLS, are experiences that relate to PTSS and are predicted by age, and experiences of internal and external shame.

In adolescence, feelings directed towards others or towards one’s self (externalising vs. internalising) is a strong predictor of being able to cope (Paulo et al., 2020). Thus, the reported experience of external and internal shame in this study related to stressful global events is a relevant finding for understanding how adolescents were coping with shame. For example, given this study was conducted during the COVID-19 pandemic, it is highly plausible that as a globally stressful and traumatic experience, the psychological impact of worrying about how they exist in the minds of others, peers, and social acceptance (external shame), may have been inadvertently captured. Consequently, our findings could reflect that the adolescents were experiencing external and internal shame as a way of directing feelings to others and about self as a way to cope with the uncertainty of COVID-19 in Australia.

Similarly, both internal and external shame were associated with traumatic events experienced directly and within the SLS role. However, both of these contexts are more closely related to the individual and are likely to elicit self-conscious emotions of shame due to the potential for perceived and actual evaluation of their social behaviour (Szentágotai-Tătar et al., 2015). The potential for adolescents to feel there is an evaluation of their social behaviour within SLS due to their role and proximity to others is amplified in the finding that shame only moderated the relationship with PTSS for PTE’s within SLS, but not for direct or global trauma.

In the emergency response context of SLS, the likelihood of feeling shame in responding to PTEs provides some implications for clinical practice and application. One implication is the additional focus and awareness of shame among young people and the likelihood of exposure to PTEs. In turn, this may inform educational and clinical interventions to increase adolescent emotional awareness in adjusting to the prevalence of PTEs within SLS. Research into adolescents has shown that those who are more open to experiences of difficult emotions are associated with high levels of emotional intelligence and effective strategies to deal with emotional issues (Castilho et al., 2017). Consequently, they can be more understanding towards themselves and able to make clearer distinctions between their emotions in order to regulate them (Mikolajczak et al., 2008). Importantly, the impact of shame on mental health outcomes, such as depression in adolescents has been shown to be mediated by self-compassion (Castilho et al., 2017). That is, helping adolescents to cope with feelings of shame through environments that frame their experience as part of common human occurrence to provide a sense of interpersonal connectedness and acceptance. Programs and interventions cultivating self-compassion in adolescents are shown to decrease depression when compared to control samples (Bluth et al., 2016). As such, self-compassion is an important regulatory process and a potential educational skill in which feelings of safeness and social affiliation and connection are antidotes for shame, self-criticism, and depression (De Rubeis & Hollenstein, 2009; Gilbert & Irons, 2008; Woods & Proeve, 2014).

While this study presents many novel findings for a previously unstudied vulnerable first responder population, there remain some considerations when interpreting the findings. First most respondents were within the non-clinical range of PTSS and therefore, the relationship between PTEs and shame in a clinical range of PTSS may highlight different results. Further studies could look at specific predictors of PTSS within a clinical subset. Similarly, the sample size (*n* = 118) represents < 1% of the 16,817 active and cadet members for the year the survey was conducted (Surf Life Saving Australia., 2021), so the findings are not fully representative of the entire cohort, but instead provide an insightful snapshot into this group. Consequently, given the diversity of the SLSA membership (and the inherent variability), we would not expect extremely strong relationships (R^2^ values > 0.50).

Age was shown to be a significant predictor of PTSS within the SLS role, whereby PTSS increased with age, which aligns with greater opportunity to be exposed to trauma, as well as older members being given more responsibilities while on patrol.

Also, these findings showed that gender (male/female) was not a significant predictor of PTSS for any trauma domain within this study. This is consistent with other research that has shown no difference between gender for indirect or direct PTEs (Wetterlöv et al., 2021). Finally, we used a broad measure of shame and adapted contexts for PTEs and therefore cannot evaluate or differentiate shame and guilt. It is possible that the association we found for internal shame and PTSS is due to an association with guilt in adolescents (Roos et al., 2014). Future research might benefit from exploring the consequences of both direct and indirect experiences of PTE’s and shame-related coping in order to develop greater understanding of potential strategies to assist.

To our knowledge this is the first paper to have examined experiences of PTEs across global, direct, and within SLS contexts in adolescent surf lifesavers. Our results suggest that adolescents within SLS report experience of PTEs and PTSS, that is moderated by shame. The intentionality of the relationship building within organisational processes after exposure to PTEs will be important in developing an ability to monitor, encourage, and motivate opportunities for skilfully settling shame, build reassuring relationships and genuinely acknowledge the complexity of coping with exposure to stressful situations.

## Data Availability

The data are not available because consent was not provided for data sharing.
